# Primary Cutaneous Gamma-Delta T-Cell Lymphoma With Long-Term Indolent Clinical Course Initially Mimicking Lupus Erythematosus Profundus

**DOI:** 10.3389/fonc.2020.00133

**Published:** 2020-02-19

**Authors:** Laura von Dücker, Mariella Fleischer, Nathalie Stutz, Markus Thieme, Mareike Witte, Detlef Zillikens, Christian D. Sadik, Patrick Terheyden

**Affiliations:** Department of Dermatology, Allergy, and Venereology, University of Lübeck, Lübeck, Germany

**Keywords:** T-cell lymphoma cutaneous, lupus erythematosus profundus/panniculitis, hemophagocytic syndrome (HPS), immunohistochemistry, cutaneous gamma-delta T-cell lymphoma

## Abstract

Primary Cutaneous Gamma-Delta (γδ) T-Cell Lymphoma (PCGDTCL) is a rare primary cutaneous lymphoma of aggressive nature. Only a few cases with an initially indolent course over years have been published. PCGDTCL can mimic diseases with benign behavior in their clinical and histopathological presentation, such as lupus erythematosus profundus, but also other lymphomas, for example subcutaneous panniculitis-like T-cell lymphoma. In our patient, the results of histopathological, immunofluorescence microscopy, and clinical examinations of early lesions first led to the diagnosis of lupus erythematosus profundus. Two years after this diagnosis and 6 years after the first clinical symptoms appeared, the disease progressed with erosive and ulcerating plaques and a PCGDTCL with hemophagocytic syndrome with an aggressive course was diagnosed. A distinct correlation of clinical, histopathological, immunohistochemical, and molecular-pathological examinations is needed to differentiate between the potentially malignant and benign diseases. Re-biopsies of different skin lesions in uncertain cases are strongly indicated. This case demonstrates that an indolent clinical phenotype can precede an aggressive clinical course in PCGDTCL.

## Introduction

Primary Cutaneous γδ T-Cell Lymphoma (PCGDTCL) is defined by a clonal proliferation of mature activated γδ T cells with a cytotoxic phenotype. It was added to the World Health Organization–European Organization for Research and Treatment of Cancer (WHO-EORTC) classification for cutaneous lymphomas as a provisional entity in 2005 and as a definitive entity in 2008 (1, 2). PCGDTCL is an extremely rare malignancy amounting to <1% of all skin lymphomas. Accordingly, only a small number of cases have been reported making PCGDTCL a poorly understood lymphoma (3–6). In those cases reported, the course of PCGDTCL was mostly short and highly aggressive with resistance to chemo- and radiotherapy (7, 8). Hemophagocytic syndrome (HPS), which is characterized by pancytopenia, fever, hepatosplenomegaly, and coagulation disturbances, may emerge in PCGDTCL. Furthermore, early ulceration of skin tumors and an involvement of the central nervous system have been reported (8–10). Immunohistochemically, most cases show a CD3^+^, CD4^−^, CD8^−^, and CD56^+^ phenotype, but CD8 expression has been reported in some patients (2, 7). The expression of cytotoxic proteins, such as granzyme B, TIA-1, and perforin, is a characteristic feature (11). The clinical and histopathological differentiation from diseases, such as the subcutaneous panniculitis-like T-cell lymphoma or lupus erythematosus profundus (LEP), may be challenging. Autoimmunity seems to be an overrepresented comorbidity (10). Here, we report a case of PCGDTCL featuring an unusual progression of disease in several aspects. Among others, in this case, PCGDTCL exhibited a protracted course of disease over years and had first been considered and treated as LEP before the disease finally exacerbated rapidly.

## Case

**Figure 1:**
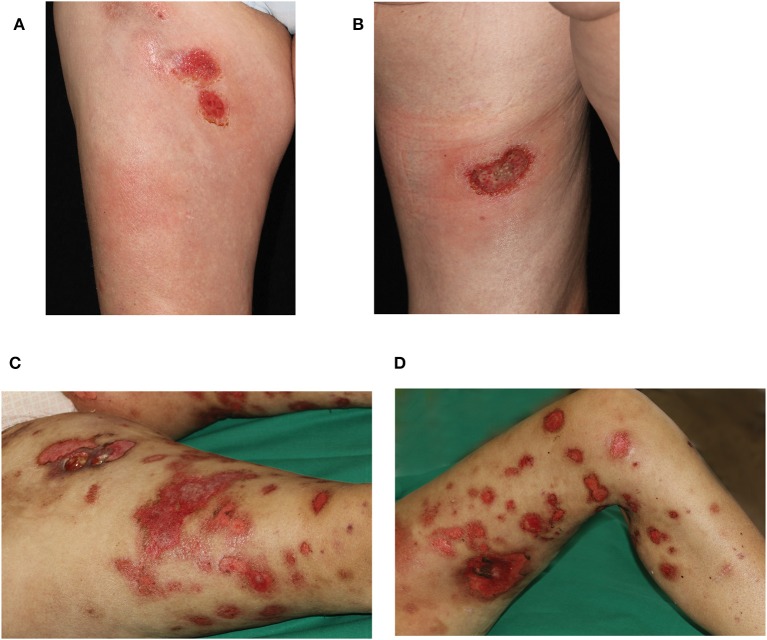
Clinical presentation during first hospitalization of the patient in our department. Erosive plaques and subcutaneous nodules **(A)** on the right ventral thigh and **(B)** the left dorsal thigh. Progressive disease 8 weeks later with disseminated erosive plaques on **(C)** right and **(D)** left leg. On the right thigh, an additional ulcer formed after biopsy of a nodule.

A 45 year-old female Caucasian consulted our hospital for the first time due to an aggravation of her known lupus erythematosus profundus (LEP). At the time of consultation, she had experienced fever for a week and had lost 6 kg of body weight within the preceding 4 weeks. Furthermore, erythematous nodules had erupted on her arms and legs, and erosions had developed spontaneously within the nodules on the thighs during the last 3 weeks (Figures 1A,B).

**Figure 2:**
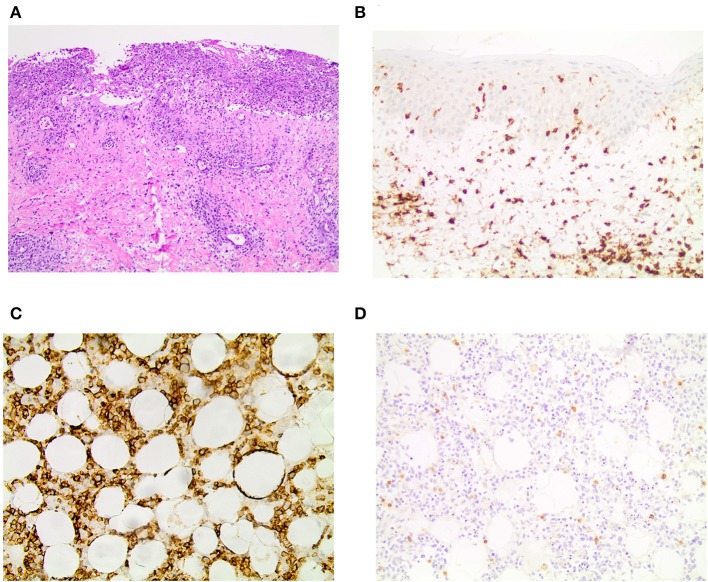
Histopathology and immunohistochemistry results. **(A)** H&E staining showing an ulceration and epidermotropism of atypical lymphocytes reaching into the dermis in 10x magnification. Immunohistochemistry for **(B)** CD3 in the skin and **(C)** CD56 in the subcutaneous fatty tissue in 20x magnification. **(D)** Beta F1 is negative in the subcutaneous fatty tissue in 20x magnification.

The patient reported that the first erythematous, tender nodules had emerged 6 years ago at her lower legs. In the beginning, the lesions did not progress and partially disappeared, so she did not consult a medical doctor. However, 2 years ago, the subcutaneous lesions disseminated quickly and also appeared on the upper legs, so the patient presented to a hospital. From the doctor's letters can be inferred that biopsies from the nodules showed histopathologically a lobular panniculitis with discrete infiltration of lymphocytes and only few histiocytes as well as focally rimming of adipocytes by neoplastic lymphocytes. Positive staining for CD3 and CD4 and to lesser extent for CD8 was seen. Additionally, the expression of the beta chain of the T-cell receptor (TCR) (beta F1) and the cytotoxic markers (TIA-1, granzyme B and perforin) was evident. However, a clonal TCR rearrangement was not detectable. Direct immunofluorescence microscopy, instead, revealed discrete band-shaped deposits of the complement factor 3 (C3), leading to the diagnosis of a LEP, although antinuclear antibodies (ANAs) were not present in the serum. There was no evidence for a systemic lupus erythematosus. A therapy with hydroxychloroquine (200–400 mg/day) and topical glucocorticosteroids was initiated and continued for ~2 years. This regimen achieved partial remission but no complete regression of lesions. When presenting with exacerbated disease at our hospital 2 years after diagnosis of LEP, routine blood work-up revealed leukopenia (2.71 × 10^9^/l; normal range: 3.9–10.2 × 10^9^/l) and elevated CRP (113 mg/l; normal range: <5 mg/l) and LDH levels (999 U/l; normal range: <250 U/l). New biopsies of one of the erosive nodules on the right thigh were taken, and the biopsied nodule subsequently swiftly started ulcerating (Figures 1C,D). Histopathology featured an interface dermatitis and atypical lymphocytes, which infiltrated the cutaneous and subcutaneous adipose tissue, and, this time, epidermotropism was also evident (Figure 2A). Lymphocytes were predominantly CD3^+^ and CD56^+^ (Figures 2B,C) as well as positive for the cytotoxic markers TIA-1, granzyme B and perforin. Additionally, the atypical lymphocytes expressed the gamma chain of the T-cell receptor. Spars CD8^+^ lymphocytes were also present. The atypical lymphocytes showed loss of CD5 and were negative for CD4. There were many admixed small CD4 positive lymphocytes in the subcutaneous fat. Beta F1 (Figure 2D) staining was negative. *In situ* hybridization showed no evidence of Epstein-Barr virus in the Epstein-Barr encoding region (EBER). At this time, a clonal TCR gamma chain rearrangement was detected, which had not been determined in the former biopsies. In accordance with the clinical presentation, PCGDTCL was diagnosed.

Nodal and visceral involvement was excluded by lymph node ultrasound, thoracic and abdominal CT scans, and bone marrow puncture. However, a splenomegaly was observed and in accordance with the laboratory findings (Table 1), and fever, a HPS was diagnosed.

**Table 1:** Summary of laboratory results key to diagnose of hemophagocytic syndrome on admission of the patient to our department.

**Laboratory value**	**Results**	**References**
Leukocytes	2.71 × 10^9^/l	3.9–10.2
Hemoglobin	8.49 g/l	12.0–15.4
Thrombocytes	99 × 10^9^/l	150–370
Ferritin	>8,000 μg/l	9–140
Fibrinogen	0.5 g/l	1.6–4.1

**Figure 3:**
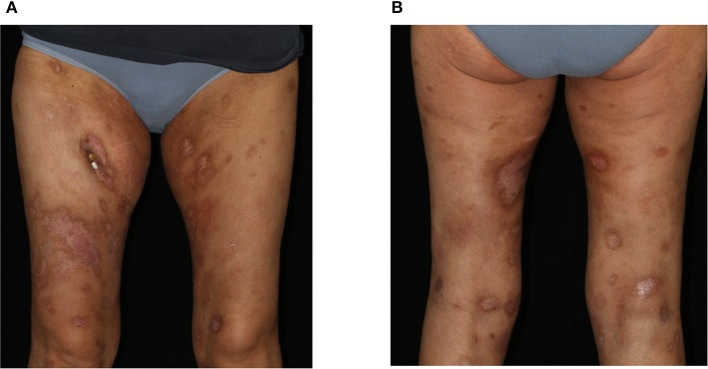
Clinical presentation after 3 cycles of CHOP therapy. Clearing of previous erosions leaving post-inflammatory hyper- and hypopigmentations on the **(A)** ventral and **(B)** dorsal aspects of both thighs.

We initiated a therapy according to the CHOP-21 regimen (cyclophosphamide, doxorubicin, vincristine, prednisolone), to which the PCGDTCL was responsive with regressing skin lesions (Figures 3A,B) as well as improvement of laboratory parameters and the patient's general condition. At the time of this report, the patient has received six cycles of CHOP therapy. However, due to a rapid relapse of both the skin nodules and the hematologic changes, a second polychemotherapy and allogenic stem-cell transplantation was performed with complete clinical remission.

## Discussion

PCGDTCL is associated with a median survival time of 15–31 months and a 5 year overall survival of 11–20% (7, 10). The case presented here, however, had been indolent for several years, before suddenly and rapidly progressing. Such a protracted course of disease has previously only been observed in exceptional cases (12–14).

This unusual clinical course further complicates the already difficult clinical and histopathological diagnosis of the disease. When progressing as slowly as in our case, the cutaneous signs of PCGDTCL resemble even more closely diverse inflammatory skin conditions, such as nodular panniculitis, pyoderma gangraenosum, erythema nodosum, and LEP (15, 16). Indeed, autoimmunological comorbidities of PCGDTCL, such as lupus erythematosus or rheumatoid arthritis, have been reported (8, 10, 11).

In most cases, PCGDTCL clinically presents with subcutaneous and infiltrated nodules and plaques, which tend to ulcerate early in the disease. The lesions are predominantly on the extremities, but lesion at other sites and dissemination are possible. In comparison, in LEP the inflammatory nodules may also appear on the face, the trunk, and the proximal extremities, but the lesions do not ulcerate (17).

The histopathological differentiation between PCGDTCL and LEP may be challenging due to missing specific PCGDTCL markers and overlapping histological patterns. This is additionally aggravated by the fact, that in some cases, the detection of lymphoma cells can be difficult due to reactive inflammation and tissue necrosis (10). Furthermore, skin biopsies, which are too small in size, not representative or superficial with missing subcutaneous tissue, can complicate the diagnosis.

Histologically, in PCGDTCL atypical lymphocytes infiltrate the skin and can present with different patterns. One or all layers of the skin may be affected, including the epidermis with epidermotropism and the dermis. Additionally, rimming of adipocytes by neoplastic lymphocytes may be seen in the subcutaneous tissue. However, these changes may also appear in other CTCLs and are therefore not specific (16, 18, 19). CD3, CD56, and cytotoxic markers (granzyme-B, TIA-1, perforin) are usually positive, and CD4 as well as the T-cell beta chain antigen receptor beta F1 are negative (10, 18). The lymphoma cells predominantly express the gamma and delta chains of the T-cell receptor (TCR). A monoclonal chain rearrangement of the TCR can be detected by polymerase chain reaction. LEP is, in contrast, histologically characterized by a lobular panniculitis of subcutaneous tissue with infiltration of plasmacytoid dendritic cells, which are often CD123^+^ (16). In our case, the histopathological picture with presence of atypical lymphocytes infiltrating the cutis and subcutaneous fat, the immunohistological pattern with detection of CD56^+^ and negative beta F1, the monoclonal rearrangement and the expression of TCR-gamma as well the clinical presentation with ulcerating nodules and the presence of fever and weight loss led to the diagnosis of PCGDTCL.

This case highlights that while clinicopathological correlation is an important aspect in the correct diagnosis of cutaneous lymphomas, in rare cases, it might be misleading. Generally, in PCGDTCL the clinical presentation with rapidly enlarging skin lesion and possible ulceration aids in the correct diagnosis. In our patient, the long-term indolent course led to an initial diagnosis of LEP, an inflammatory disease which can exhibit a histopathological pattern similar to that of cutaneous lymphomas located in the subcutaneous tissue. Particularly in the light of unusual clinical presentations with a protracted course and localized slowly growing tumors, a repeated correlation between clinical, histopathological, immunohistochemical, and molecular pathological features might be necessary. Additionally, one may raise the question, if these rare cases represent a distinct variant of PCGDTCL. Our case underlines the challenge of diagnosing a PCGDTCL as the clinical presentation and the diagnostic findings can be misleading and indolent courses over years may be possible. Although PCGDTCL is a rare disease, patients with panniculitis-like lesions, who do not respond to therapy, should be closely monitored, and repeated biopsies should be performed during the course of the disease to establish the correct diagnosis.

## Ethics Statement

Written informed consent was obtained from the patient for the publication of any potentially identifiable images or data included in this article.

## Author Contributions

LD, CS, and PT conceived of the presented idea and wrote the manuscript with support from all other authors. MF and NS carried out the histological examinations. DZ supervised the findings of this work. All authors discussed the results and contributed to the final manuscript.

### Conflict of Interest

PT: speaker's honoraria from BMS, Novartis, MSD, Pierre-Fabre, CureVac and Roche, consultant's honoraria from BMS, Novartis, Pierre-Fabre, Merck Serono, Sanofi und Roche and travel support from BMS, Pierre-Fabre and Roche. The remaining authors declare that the research was conducted in the absence of any commercial or financial relationships that could be construed as a potential conflict of interest.
